# Global Learner Feedback on Continuing Medical Education–Accredited e-Learning Modules in Pediatric Endocrinology and Diabetes: Cross-Sectional Study

**DOI:** 10.2196/67332

**Published:** 2026-01-30

**Authors:** Yvonne G van der Zwan, Conny van Wijngaard-de Vugt, Abdulsalam Abu-Libdeh, Evangelia Kalaitzoglou, Zacharoula Karabouta, Stenvert L S Drop, Annemieke M Boot, Sze May Ng, Jan Idkowiak

**Affiliations:** 1 Department of Paediatrics, Isala, Zwolle, The Netherlands; 2 WV Research, Advice and Management, Brunssum, The Netherlands; 3 Department of Pediatrics, Division of Pediatric Endocrinology, Makassed Hospital and Al-Quds Medical School, Jerusalem, Palestinian Territory, Occupied; 4 Division of Pediatric Endocrinology, Hadassah University Medical Center & Hadassah Medical School, Jerusalem, Israel; 5 Department of Pediatrics, University of Kentucky, Barnstable Brown Diabetes Center, Lexington, KY, United States; 6 2nd Paediatric Department, AHEPA University General Hospital, Thessaloniki, Greece; 7 Division of Endocrinology, Department of Pediatrics, Sophia Children’s Hospital, Erasmus MC, Rotterdam, The Netherlands; 8 Department of Pediatric Endocrinology, University Medical Center Groningen, University of Groningen, Groningen, The Netherlands; 9 Edge Hill University, Ormskirk, England, United Kingdom; 10 Department of Pediatric Endocrinology, Mersey and West Lancashire Teaching Hospitals NHS Trust, Southport, United Kingdom; 11 Department of Metabolism and Systems Science, College of Medicine and Health, University of Birmingham, Edgbaston, IBR tower, level 2, Birmingham, B15 2TT, United Kingdom, 44 1214152764; 12 Centre for Endocrinology, Diabetes and Metabolism, Birmingham Health Partners, University of Birmingham, Birmingham, United Kingdom; 13 Department of Endocrinology and Diabetes, Birmingham Children’s Hospital, Birmingham Women’s and Children’s NHS Foundation Trust, Birmingham, United Kingdom

**Keywords:** e-learning, continuing medical education, endocrinology, European Society for Paediatric Endocrinology, ESPE, International Society for Pediatric and Adolescent Diabetes, ISPAD, pediatric endocrinology, pediatrics, diabetes, eHealth, digital health, medical education

## Abstract

**Background:**

The European Society for Paediatric Endocrinology (ESPE) e-Learning wesite is a free, globally accessible online resource to enhance learning in pediatric endocrinology and pediatric diabetes. The content is created by world-leading experts in pediatric endocrinology and pediatric diabetes and is closely aligned with published international consensus guidelines. In August 2022, 30 hours of e-learning courses received accreditation from the European Accreditation Council for Continuing Medical Education (CME). These CME courses cover three categories: (1) pediatric endocrinology, (2) pediatric diabetes, and (3) pediatric endocrinology in resource-limited settings.

**Objective:**

This study aimed to assess learners’ demographics and feedback from mandatory surveys after completion of CME e-learning courses and to identify areas for improvement.

**Methods:**

The ESPE e-learning committee created a mandatory survey for each CME e-learning module. The survey includes baseline demographics and feedback on the quality of the learning content, assessed using a 5-point Likert scale. Data were extracted from the start of the CME modules in August 2022 until September 2025.

**Results:**

A total of 567 surveys were completed: 286 (50.4%) in the category pediatric endocrinology, 225 (39.7%) in the category pediatric diabetes based on the International Society for Pediatric and Adolescent Diabetes guidelines, and 56 (9.9%) in the category pediatric endocrinology in resource-limited settings. There was global participation, with most learners practicing in Europe (n=333, 59%), followed by Asia (n=124, 22%), Africa (n=53, 9%), the Americas (North America, n=45, 8%; and South America, n=11, 2%), and Oceania (n=1, 0%). Most of the users indicated that they were medical experts (n=210, 37%), followed by fellows or residents (n=223, 39%), and medical students and nurses (n=29, 5% and n=32, 6%, respectively); overall, 10% (n=56) of learners practice in resource-limited countries. Overall, the learning content was well received for all modules regarding accessibility, organization, level of interest, improvement of learners’ clinical practice, appropriateness of content, and provision of feedback (median Likert score 4, IQR 4-5). Learners’ free-text feedback identified some areas for improvement, including reducing text-heavy content and providing more graphical content and more interactive case reports. Most learners’ free-text feedback consisted of encouraging and thankful comments.

**Conclusions:**

The ESPE CME–accredited e-learning modules are well received, providing globally free CME education in pediatric endocrinology and pediatric diabetes. These findings support the continued development and promotion of open-access CME platforms, with the aim of improving global equity in specialist medical education and focusing on educational impact.

## Introduction

The European Society for Paediatric Endocrinology (ESPE) e-Learning website [1] is a free, globally accessible online tool to enhance learning in pediatric endocrinology (PE) and pediatric diabetes (PD) worldwide [2-4].

The ESPE e-Learning website was first published online in 2012. Since then, the content and use have expanded with courses available in PE and PE in resource-limited settings (RLSs). The e-learning committee of ESPE and the International Society for Pediatric and Adolescent Diabetes (ISPAD) are collaboratively responsible for establishing and maintaining the e-learning platform, as well as ensuring the development and quality of its content. This freely accessible online portal allows medical students, fellows, specialists, nurses, and tutors from around the world to share, contribute to, and expand their knowledge through interactive chapters and case-based learning, covering both core- and advanced-level modules [5,6]. During the COVID-19 pandemic, e-learning became an essential resource for continuous professional development, and online learning demonstrated that the effects of e-learning are equivalent to traditional learning [7-9]. e-Learning has now become a solution for overcoming training barriers posed by social distancing rules, offering education and learning opportunities to students, trainees, and physicians to help them maintain essential competencies and continue professional development. It is especially beneficial for individuals working residential shifts with work hour restrictions, which frequently conflict with in-person attendance at didactic lectures, and for those living in remote areas.

The ESPE e-learning website contains 80 chapters and more than 130 cases covering core and advanced learning courses on the most common endocrine disorders, including diabetes; of these, 16 chapters and 25 cases target health care professionals based in RLSs. The content includes normal physiology, pathophysiological mechanisms underlying endocrine diseases, diagnostic approaches or algorithms, and management based on international expert consensus and published guidelines. Direct feedback on questions from users is encouraged. Recently, two video masterclasses delivered by internationally recognized experts in the field have been added. Steering, conceptual, and content oversight is provided by the ESPE-ISPAD e-learning committee, which is responsible for creating and maintaining the e-learning website, ensuring up-to-date information and high-quality standards.

All courses are available in English, and all courses in the category *health care in resource-limited settings*, which is specifically intended for practitioners working in primary, secondary, and tertiary health care centers in resource-limited countries, are available in four additional languages (ie, French, Spanish, Swahili, and Chinese) [10].

Since August 2022, 30 credit hours of ESPE-ISPAD continuing medical education (CME) e-learning courses created by world-leading experts in PE and PD have received accreditation from the European Accreditation Council for CME (EACCME). These CME courses are typically based on consensus guidelines. They are organized in three categories with 10 courses each in the categories *pediatric endocrinology*, *pediatric diabetes,* and *health care in resource-limited settings*. The latter module is available in five different languages (ie, English, French, Spanish, Swahili, and Chinese). Each CME course takes approximately 1 to 1.5 hours of educational time, and completion of a course provides one European CME credit.

To promote global awareness and uptake of the CME-accredited modules, the ESPE e-learning platform was actively disseminated through multiple channels. These included presentations at international society meetings, such as ESPE and ISPAD, regular features in quarterly ESPE newsletters and bimonthly ISPAD newsletters, and targeted outreach via social media platforms, including Facebook and X. These efforts aimed to ensure broad visibility and accessibility of the platform across diverse geographic and professional audiences.

To date, little is known about how these accredited modules are used and perceived by health care professionals globally. Herein, we aimed to evaluate the geographical reach, user demographics, and learner feedback on the accredited CME modules, specifically examining how health care professionals from diverse regions and professional backgrounds engage with and evaluate the CME-accredited modules. Therefore*,* we conducted a descriptive cross-sectional analysis of the mandatory postcourse survey data collected between August 2022 and September 2025. We hypothesized that the modules would be positively received across user groups and that learners would identify specific areas for improvement. The findings may help inform educators, module developers, and professional societies in improving their approach to expand equitable access to high-quality, guideline-based CME in PE and PD.

## Methods

### Ethical Considerations

Participation in the feedback survey was entirely voluntary, and informed consent was implied by completion of the survey. Respondents were clearly informed that they could opt out at any time by not submitting the survey. No additional consent was required because this was a secondary analysis of anonymized data collected under the original consent process for CME module participation, which included agreement to provide feedback for service evaluation purposes.

Ethical approval was not deemed necessary as the analysis of the survey was conducted as an evaluation and quality improvement exercise to evaluate the educational offering. Data were collected anonymously from adults/professionals (no vulnerable population); no identifiable information was collected; there was no intervention, randomization, or direct implications on patient care; and the participation was entirely voluntary.

No financial or material compensation was provided to participants for completing the survey.

### Survey Design and Setting

The reporting of this study adhered to the CHERRIES (Checklist for Reporting Results of Internet E-Surveys) to ensure methodological transparency and reproducibility [11]. The checklist was applied to all stages of the online evaluation process, including survey design, delivery, and data handling. The evaluation questionnaire was developed by the ESPE e-learning team and pretested with a small group of users to assess clarity, face validity, and technical functionality. The final version was implemented within the online education platform, which automatically invited participants to complete the anonymous feedback survey upon module completion (items 5‐8). Participation was mandatory upon completion of the course; no personal data, as per General Data Protection Regulation requirements, were collected. No incentives were offered. Measures to prevent multiple submissions included limiting one response per user account and restricting survey access to registered participants only. Data completeness checks were built into the survey logic, and incomplete responses were excluded from the main analysis but retained for sensitivity assessment. All data were collected through a secure institutional server with encrypted transmission and stored in accordance with international data governance standards.

The survey was created by the ESPE e-learning committee to obtain information on learners’ demographics and to evaluate the use and quality of the courses. The content, in accordance with Kirkpatrick’s evaluation type level 1 (*reaction*) [12,13], included questions about learners’ professional backgrounds and countries of residence. Feedback on the quality of the learning content, presentation, accessibility, and the anticipated impact on clinical practice was assessed using a 5-point Likert scale, ranging from strongly agree (Likert scale=1), agree (Likert scale=2), neutral (Likert scale=3), disagree (Likert scale=4), and strongly disagree (Likert scale=5). We used a different Likert scale for one specific question asking for the degree of difficulty of quiz questions provided in the modules (Likert scale 1=too easy, 2=easy, 3=appropriate, 4=difficult, and 5=too difficult). Likert scales were chosen to allow nuanced assessment of attitudes and perceptions, are well understood by respondents, and facilitate both descriptive and inferential statistical analyses. Additional feedback on content, user interface, or other issues was encouraged through an open free-text question. The detailed survey is provided in Multimedia Appendix 1. Data were analyzed from the launch of the CME module in August 2022 until September 2025. The professional background was categorized based on professional training and experience in PE and grouped as (1) medical expert or consultant, (2) fellow, (3) resident, (4) medical student, (5) nurse, and (6) other (free text). Health care officers from research-limited countries were asked if they were based in primary, secondary, or tertiary level settings to understand resources and facilities available (primary level: basic or rural with very limited laboratory and imaging facilities; secondary level: district or regional hospitals with limited laboratory and imaging facilities; and tertiary level: main or national referral hospitals with most but not all laboratory and imaging facilities [10]).

### Visualization of Global Participation

Geographical distribution of participants completing the online CME modules was visualized using *Plotly* (version 5.24.0; Plotly Technologies Inc). Country names from the registration dataset were matched to International Organization for Standardization–recognized country centroids, and each country was represented by a proportional bubble plotted on a world map in a natural-earth projection.

### Statistical Analysis

Descriptive statistics were applied. When analyzing the 5-point Likert scale, we used the median (IQR) as appropriate for an ordinal scale; however, we have also provided the mean and SD to describe the level of dispersion. As Likert responses are ordinal and nonnormally distributed, comparisons across the three modules were performed using the Kruskal-Wallis H test. Where relevant, *P* values <.05 were considered statistically significant. A stacked Likert plot was generated to visualize the proportional distribution of responses across all survey questions. Free-text feedback was independently coded into four categories by two authors. Discrepancies were discussed, and final classifications were reached by consensus. We have used Microsoft Excel and GraphPad Prism for descriptive statistics and graphical illustration of the data.

## Results

From August 2022 to September 2025, a total of 567 surveys were completed. Of those, 286 (50.4%) were in the category pediatric endocrinology, 225 (39.7%) were in the category pediatric diabetes, and 56 (9.9%) were in the category pediatric endocrinology in RLS.

### Learner’s Background

**Figure 1.**
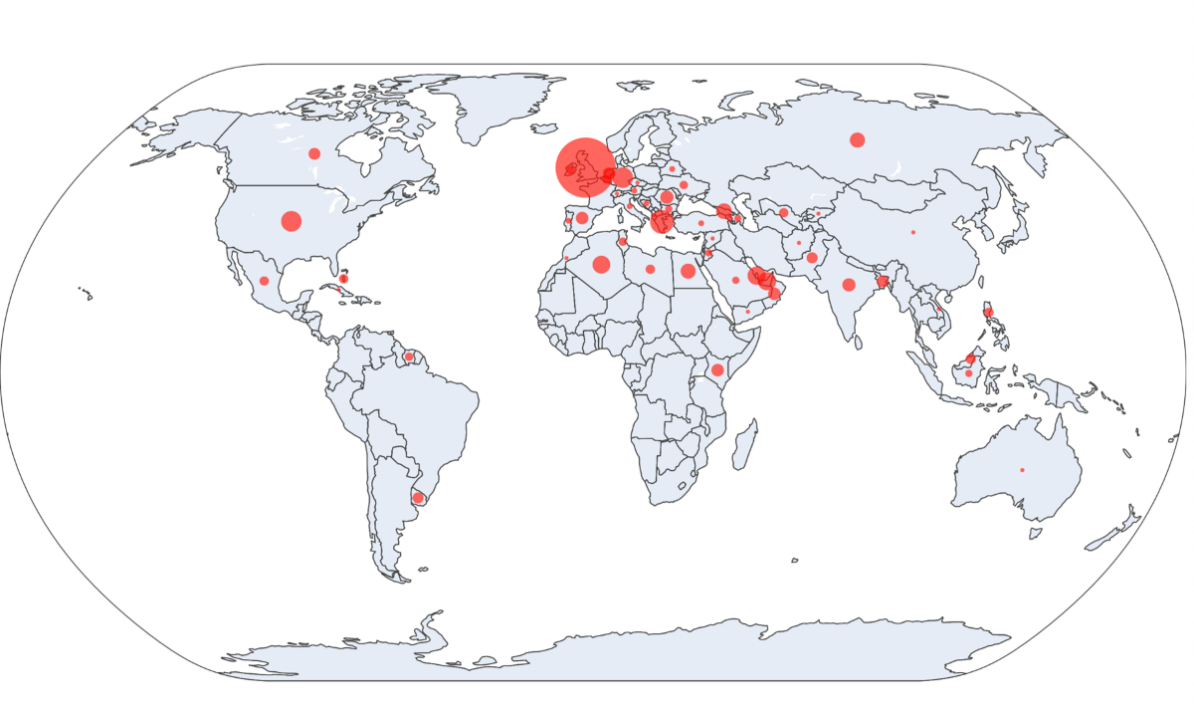
Global distribution of participants completing online continuing medical education modules***.*** Each bubble represents a country, with bubble size proportional to the number of completed modules. Red semitransparent bubbles are projected onto a natural-earth world map, with representative bubble sizes (n=5, 10, and 100) shown for scale.

There was global participation, but most learners were practicing in Europe (n=333, 59%), followed by Asia (n=124, 22%), Africa (n=53, 9%), the Americas (North America, n=45, 8%; and South America, n=11, 2%), and Oceania (n=1, 0%; Figure 1 and Multimedia Appendix 2). Although the RLS courses are available in Mandarin, there was no participation from China.

**Figure 2.**
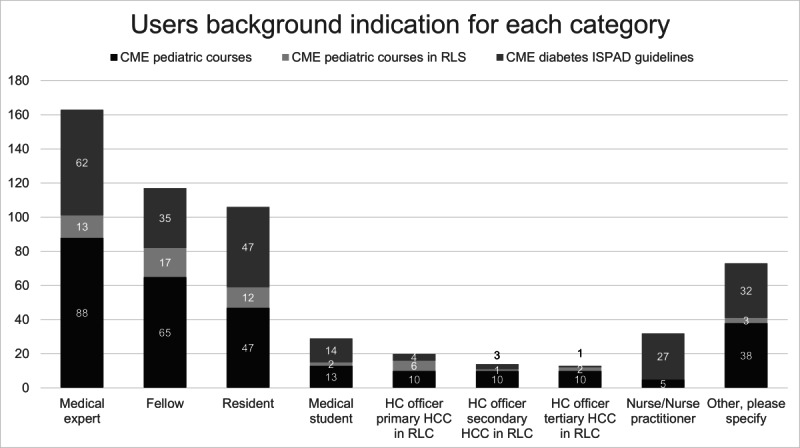
Representation of learners according to training background. CME: continuing medical education; HC: health care; HCC: health care center; ISPAD: International Society for Pediatric and Adolescent Diabetes; RLS: resource-limited setting.

Most users indicated that they were medical experts (210/567, 37%), followed by fellows or residents (223/567, 39%). Medical students and nurses formed 5% and 6%, respectively, of the total group, and 13% were categorized under *other* (Figure 2). Relevant specifications given in the category *other* include lecturer, psychologist, patient advocate, diabetes educator, nutritionist, and education support worker.

Twelve percent (n=70/567) of learners practice in RLS countries, of which 20 work as health care officers in primary health care centers, 14 work in secondary health care centers, and 13 work in tertiary health care centers [14].

### Feedback on CME Modules

**Figure 3.**
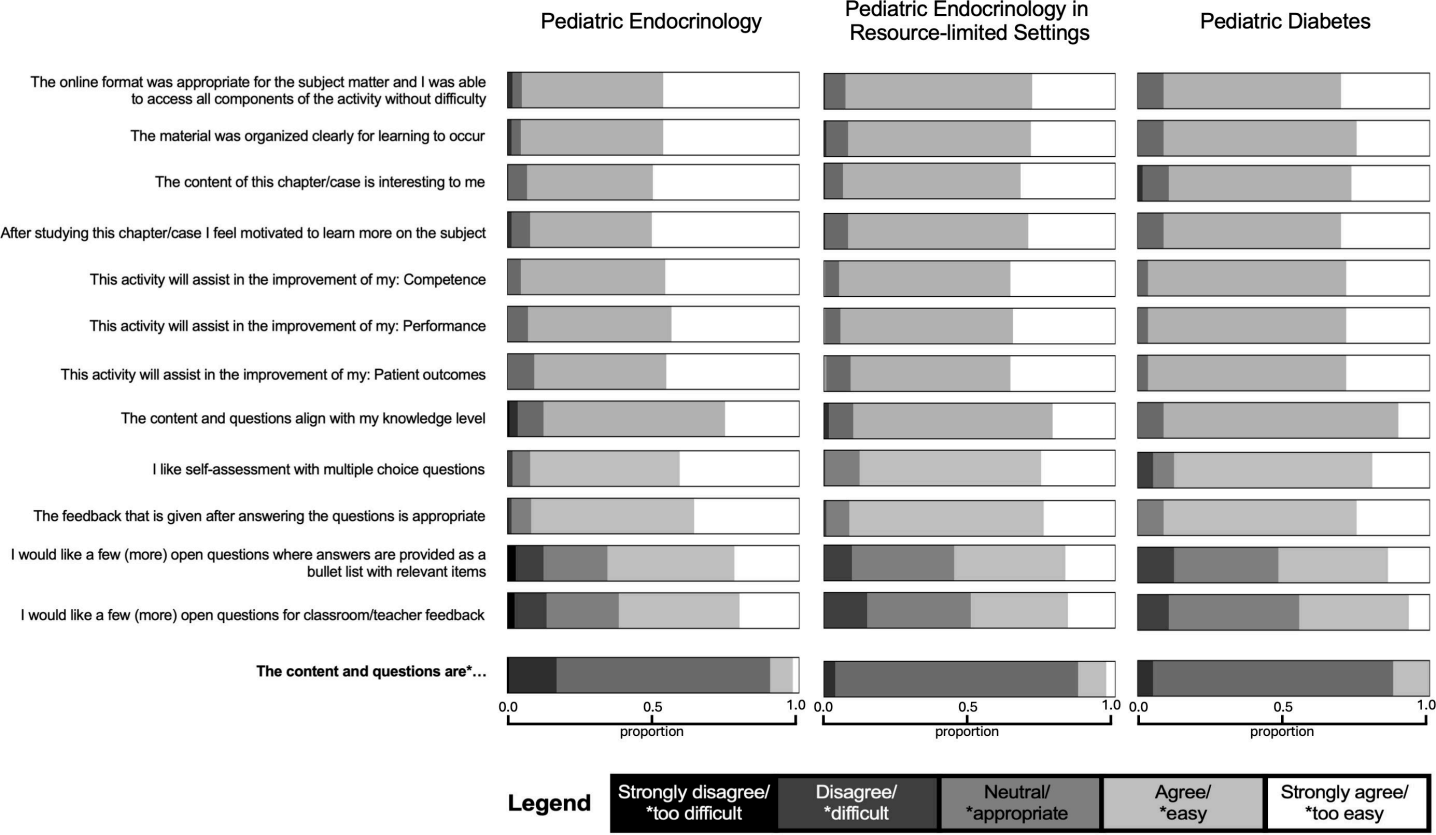
Stacked Likert distribution of responses across all survey questions. This figure displays the proportional distribution of Likert-scale responses, separate for each module (pediatric endocrinology, pediatric endocrinology in resource-limited settings, and pediatric diabetes). Each horizontal bar represents one survey question, subdivided into gray-shaded coded segments corresponding to the five Likert response categories. Segment widths reflect the proportion of total responses in each category. The plot illustrates the overall positive skew of responses across the curriculum, with most items demonstrating high levels of agreement (Likert 4‐5*)*, while specific questions show greater dispersion, indicating more heterogeneous participant views.

Across all survey items, overall learner satisfaction was high, with most responses falling within the *agree* or *strongly agree* categories (Table 1, Figure 3). The median scores for the majority of questions were 4.0, with means consistently above 4.0 for questions relating to content quality, clarity, motivation, competence, and perceived educational value. The feedback on the questions’ difficulty level was also narrow, with the majority of responses falling into the *appropriate* category. The PE module generally received the highest mean ratings across items, whereas the RLS and PD modules showed similar, though slightly lower, response patterns. IQRs were narrow for most questions, indicating highly compressed distributions with limited variability. Kruskal-Wallis testing revealed statistically significant differences between modules for most survey items (*P*<.05), primarily due to a higher proportion of strongly positive responses in the PE module. Exceptions included the question on perceived improvement in performance, where module differences were not statistically significant. Items relating to preferences for additional open-ended questions (eg, bullet list and teacher feedback formats) received comparatively lower mean scores and showed greater variability. Overall, the findings indicate strong learner engagement and perceived educational value, with some variation in response patterns across modules (Table 1, Figure 3).

**Table 1.** Descriptive and comparative statistics for all Likert-scale survey questions across the three modules (pediatric endocrinology [PE], n=283; PE in resource-limited settings [RLS], n=56; and pediatric diabetes [PD], n=228).

Survey question^a^ and modules	Likert score responses, n					Comparative statistics		
	1	2	3	4	5	Median (IQR)	Mean (SD)	*P* value
The online format was appropriate for the subject matter and I was able to access all components of the activity without difficulty.^b^								*<.001*
PE	0	5	9	137	132	4.0 (4.0-5.0)	4.40 (0.64)	
RLS	0	0	5	34	17	4.0 (4.0-5.0)	4.21 (0.59)	
PD	0	1	16	146	65	4.0 (4.0-5.0)	4.21 (0.58)	
The material was organized clearly for learning to occur.^b^								*<.001*
PE	0	4	9	138	132	4.0 (4.0-5.0)	4.41 (0.63)	
RLS	0	0	5	37	14	4.0 (4.0-4.2)	4.16 (0.57)	
PD	0	2	17	143	66	4.0 (4.0-5.0)	4.20 (0.60)	
The content of this chapter or case is interesting to me.^b^								*<.001*
PE	0	1	18	122	142	5.0 (4.0-5.0)	4.43 (0.63)	
RLS	0	1	5	35	15	4.0 (4.0-5.0)	4.14 (0.65)	
PD	0	1	14	139	74	4.0 (4.0-5.0)	4.25 (0.58)	
After studying this chapter or case, I feel motivated to learn more on the subject.^b^								*<.001*
PE	0	4	18	118	143	5.0 (4.0-5.0)	4.41 (0.68)	
RLS	0	0	5	34	17	4.0 (4.0-5.0)	4.21 (0.59)	
PD	0	1	18	141	68	4.0 (4.0-5.0)	4.21 (0.59)	
This activity will assist in the improvement of my competence.^b^								*.02*
PE	0	0	13	140	130	4.0 (4.0-5.0)	4.41 (0.58)	
RLS	0	0	2	38	16	4.0 (4.0-5.0)	4.25 (0.51)	
PD	0	1	11	134	82	4.0 (4.0-5.0)	4.30 (0.58)	
This activity will assist in the improvement of my performance.^b^								.12
PE	0	0	20	139	124	4.0 (4.0-5.0)	4.37 (0.61)	
RLS	0	0	2	38	16	4.0 (4.0-5.0)	4.25 (0.51)	
PD	0	1	12	135	80	4.0 (4.0-5.0)	4.29 (0.58)	
This activity will assist in the improvement of my patient outcomes.^b^								*<.001*
PE	0	0	26	128	129	4.0 (4.0-5.0)	4.36 (0.65)	
RLS	0	0	2	38	16	4.0 (4.0-5.0)	4.25 (0.51)	
PD	0	2	19	125	82	4.0 (4.0-5.0)	4.26 (0.64)	
The content and questions align with my knowledge level.^b^								*<.001*
PE	2	8	25	176	72	4.0 (4.0-5.0)	4.09 (0.72)	
RLS	0	0	5	45	6	4.0 (4.0-4.0)	4.02 (0.45)	
PD	0	4	19	156	49	4.0 (4.0-4.0)	4.10 (0.60)	
I like self-assessment with multiple choice questions.^b^								*.02*
PE	0	5	17	145	116	4.0 (4.0-5.0)	4.31 (0.67)	
RLS	0	3	4	38	11	4.0 (4.0-4.0)	4.02 (0.70)	
PD	0	1	27	142	58	4.0 (4.0-5.0)	4.13 (0.61)	
The feedback that is given after answering the questions is appropriate.^b^								*<.001*
PE	1	3	19	158	102	4.0 (4.0-5.0)	4.26 (0.66)	
RLS	0	0	5	37	14	4.0 (4.0-4.2)	4.16 (0.57)	
PD	0	2	18	152	56	4.0 (4.0-4.0)	4.15 (0.58)	
I would like a few (more) open questions where answers are provided as a bullet list with relevant items.^b^								*<.001*
PE	8	27	62	123	63	4.0 (3.0-4.0)	3.73 (1.00)	
RLS	0	7	20	21	8	4.0 (3.0-4.0)	3.54 (0.89)	
PD	0	22	80	87	39	4.0 (3.0-4.0)	3.63 (0.88)	
I would like a few (more) open questions for classroom/teacher feedback.^b^								*.02*
PE	7	31	70	117	58	4.0 (3.0-4.0)	3.66 (1.00)	
RLS	0	6	25	21	4	3.0 (3.0-4.0)	3.41 (0.78)	
PD	0	34	81	76	37	3.0 (3.0-4.0)	3.51 (0.94)	
The content and questions are:^c^								*<.001*
PE	2	46	207	22	6	3.0 (3.0-3.0)	2.94 (0.59)	
RLS	0	3	46	7	0	3.0 (3.0-3.0)	3.07 (0.42)	
PD	0	9	190	22	7	3.0 (3.0-3.0)	3.12 (0.50)	

a For each question, response frequencies on a 5-point Likert scale were expanded into individual-level datasets to compute descriptive statistics, including total responses, median, mean, IQR, and SD. Differences in score distributions across modules were assessed using the Kruskal-Wallis H test, appropriate for ordinal, nonnormally distributed data. *P* values <.05 were considered statistically significant and are indicated by italics. Higher scores reflect more favorable evaluations.

b Likert scale responses: 1=strongly disagree; 2=disagree; 3=neutral; 4=agree; and 5=strongly agree.

c Likert scale responses: 1=too easy; 2=easy; 3=appropriate; 4=difficult; and 5=too difficult.

Not all participants provided free-text feedback. Table 2 illustrates the distribution of free-text feedback received, categorized into four main categories, with the largest being various forms of appraisal. Improvement suggestions, such as requesting more interactive cases and algorithms, are encouraging. Layout issues are helpful to guide improvements, and similarly, critical comments about the difficulty of the content and excessive text are incentives to indicate more clearly the level of the course (core vs advanced), and replacing text-heavy chapters with bullet styles using artificial intelligence diligently.

**Table 2.** Free-text feedback from completed continuing medical education modules.

Domains	Overall (N=384), n (%)	PE^a^ (n=197), n (%)	RLS^b^ (N=26), n (%)	PD^c^ (n=161), n (%)	Example comments
Praise or approval	274 (71.3)	145 (73.6)	21 (80.8)	108 (67.1)	Content is appropriate, good, excellent, perfect, helpful, informative, useful, adds knowledge and scientific thinking
Content suggestions	47 (12.2)	27 (13.7)	3 (11.5)	17 (10.6)	Needs more interactive cases, more algorithms, more detailed information, better explanations of multiple-choice answers, and more video content
Layout suggestions	28 (7.3)	13 (6.6)	2 (7.7)	13 (8.1)	Buttons need to be bigger, multiple replies in multiple-choice questions unclear, minimize menu left side of screen, and typos and missing sentences
Criticism or disapproval	35 (9.1)	12 (6.1)	0	23 (14.2)	Slightly *dry*, too difficult, too much text, too many references, and inappropriate for my work setting

a PE: pediatric endocrinology.

b RLS: PE in resource-limited setting.

c PD: pediatric diabetes.

## Discussion

In an era where equitable access to CME is increasingly important, this study highlights the global uptake and positive reception of the ESPE CME–accredited e-learning modules made freely available in PE and PD, particularly among professionals in both high-resource and RLSs.

### Principal Findings

Over a 3-year period, 567 learners from five continents completed postcourse surveys. The majority of participants were medical experts or trainees. Encouragingly, there were also learners practicing in RLSs. Feedback was overall very positive, with the majority of responses scoring 4 or 5 on a 5-point Likert scale. Free-text feedback suggested areas for improvement, including reducing text-heavy content and increasing interactivity and visual elements.

### Implications of Findings

These findings support the value of free, guideline-based CME resources in PE and PD, particularly in expanding access to continuing education globally. The content is not only in line with the training requirements in PE and PD [15] but is also complementary to the requirements of the neonatology training curriculum [16]. Moreover, it is relevant for internists dealing with former pediatric patients with chronic congenital or acquired pediatric disorders [17].

A prerequisite of self-directed learning is that learners are internally motivated to take responsibility for their learning through a process in which they identify their own learning needs, use a variety of resources to meet these needs, and evaluate their learning to ensure that their learning needs have been met [18].

The high satisfaction ratings suggest that the ESPE platform meets these self-directed learning needs of a diverse professional audience. The engagement of health professionals, such as nurses and dietitians, also reflects the growing multidisciplinary nature of pediatric endocrine care. The feedback received provides actionable insights to improve the user experience and tailor content to different learning styles and clinical contexts.

Learners’ free-text feedback has provided actionable insights for improving the ESPE e-learning modules. Common suggestions, such as adding more interactive cases, in particular when content is text-heavy; simplifying algorithms; and reducing text density, have informed content updates. The ESPE e-Learning website is moving to a new online platform, which allows for more visually engaging content and the use of artificial intelligence, which we anticipate will enhance learners’ experience, engagement, and accessibility across varying levels of training. Categorizing feedback into themes also enables the committee to track trends and evaluate the impact of changes over time, ensuring the platform evolves in response to user needs.

### Comparison With Prior Work

The EACCME implemented criteria for the accreditation of e-learning materials in 2009 and conducted an audit of CME–-Continuous Professional Development (events between 2017 and 2019), which included 385 e-learning materials [19]. However, no details on the assessment of these e-learning materials are provided, and studies focusing on accreditation of self-directed interactive asynchronous online learning designs are scarce [20,21]. Blomberg et al [22] highlighted a post-COVID-19 pandemic shift toward hybrid and digital CME formats, emphasizing accessibility and learner-centered design. Curran et al [20] concluded that an accredited, asynchronous e-learning module provides flexibility, accessibility, and scalability, particularly for geographically dispersed health professionals and described a structured approach to developing accredited e-learning modules, underscoring the importance of stakeholder engagement and iterative content development. Additionally, Tudor Car et al [23] demonstrated that digital problem–based learning is as effective as traditional methods for knowledge acquisition and may be superior for skill development.

Our results are consistent with earlier evaluations of the ESPE platform. Ng et al [4] reported sustained global engagement with the nonaccredited modules over a 10-year period. Kalaitzoglou et al [10] demonstrated the effectiveness of multilingual modules in improving access in RLSs, which aligns with our finding that 9% of users came from such contexts. Prior studies by Drop et al [5] and Kranenburg-van Koppen et al [6] emphasized the importance of interactive, case-based learning, a preference echoed in our learners’ feedback. Our study adds to this literature by focusing specifically on the CME-accredited modules and providing structured postcourse feedback data.

This supports the notion that digital CME platforms, such as the ESPE e-learning portal, are well positioned to meet evolving global educational needs in PE and PD.

### Strengths and Limitations

A key strength of this study is its global scope and inclusion of diverse professional roles, offering a comprehensive view of how the CME modules are used and perceived. The survey’s mandatory nature ensured a high response rate. However, the study has limitations. It relies on self-reported data, which may be subject to response bias. The absence of pre- and post-module assessments limits our ability to measure learning outcomes objectively. Additionally, participation from certain regions (eg, China, Brazil, and Scandinavia) was lacking. Several factors may explain the lack of participation from certain regions. First, national CME accreditation policies vary widely; some countries may not recognize EACCME credits or may not require CME for specialist revalidation, reducing the incentive to engage with external platforms. Second, language barriers may persist despite the facilitating multilingual content, particularly if promotional materials or user interfaces are not localized. Third, awareness and visibility of the platform may be limited in regions where ESPE or ISPAD has fewer members. Fourth, cultural preferences for in-person or locally developed educational resources may influence uptake. Finally, technological access and digital literacy may still pose challenges in some areas, particularly in rural or underserved settings [24]. These insights underscore the need for targeted outreach strategies, local ambassadors, and region-specific promotional campaigns to improve awareness and adoption. Several authors emphasize the importance of partnerships with international societies, such as ESPE and ISPAD, and the support by their organizational infrastructure as of critical importance [4,22,25,26].

The accreditation credit points (European CME credit) are based on educational time spent and not on educational impact. In several papers, transitioning CME crediting from being time based to impact based is advocated with a plea for more rigorous quantitative or qualitative assessments. The assessment in the format of surveys should be appropriate to the intended goals or outcomes of the accredited education, measuring improvements in learner knowledge, skills, and competencies, professional performance, and ultimately in changes in patient health status [19,27,28]. The International Academy for Continuing Professional Development Accreditation created a shared set of international standards for accrediting CME [28]. De Leeuw et al [27] constructed a validated 7-step Medical E-Learning Evaluation Survey. The authors conclude that the Medical E-Learning Evaluation Survey is useful and understandable, adding value for e-learning creators. However, it is impossible to predict how motivated users will be to provide useful feedback. Therefore, a survey should always be accompanied by an in-depth focus group evaluation with the users. The adoption of international standards would establish a global framework, and substantive equivalency would enable international collaboration, reciprocity of credits, and broader access to high-quality education for health care professionals [28].

To enhance the platform’s impact, future efforts should focus on developing pre- and postmodule assessments to evaluate knowledge gain, increasing interactivity through multimedia content and case simulations, strengthening outreach in underrepresented regions by partnering with local societies and ambassadors, and exploring the long-term impact of the modules on clinical practice and patient outcomes. The ESPE-ISPAD e-Learning platform has the potential to serve as a model for other specialties seeking to deliver equitable, high-quality CME globally.

### Conclusions

The ESPE-ISPAD CME–accredited e-learning modules are widely accessed and well received by health care professionals globally, offering free, high-quality education in PE and PD. Moreover, the asynchronous self-paced designs afford greater convenience and flexibility for providers in accessing the CME courses at times that are best for them [26].

Learners reported high satisfaction with the content and delivery, and feedback highlighted opportunities to further enhance interactivity and accessibility.

These findings support the continued development and promotion of open-access CME platforms to improve global equity in specialist medical education. Ongoing efforts are needed to expand outreach, refine content based on user feedback, and make accreditation more dynamic, evidence-based, and focused on educational impact.

## Supplementary material

10.2196/67332 Multimedia Appendix 1 Content of the continuing medical education feedback survey.

10.2196/67332 Multimedia Appendix 2 Continuing medical education courses completed.

## Abbreviations

CHERRIES: Checklist for Reporting Results of Internet E-Surveys
CME: continuing medical education
EACCME: European Accreditation Council for Continuing Medical Education
ESPE: European Society for Paediatric Endocrinology
ISPAD: International Society for Pediatric and Adolescent Diabetes
PD: pediatric diabetes
PE: pediatric endocrinology
RLS: resource-limited setting
