# Supplementary material for: Global Learner Feedback on Continuing Medical Education–Accredited e-Learning Modules in Pediatric Endocrinology and Diabetes: Cross-Sectional Study
Source: JMIR Med Educ. 2026 Jan 30;12:e67332. doi: 10.2196/67332 (PMC12857890; doi:10.2196/67332)
Supplement: Multimedia Appendix 1 [file mededu-v12-e67332-s001.docx]

**Supplementary Appendix 1**

**Content of the CME Feedback Survey**

**User group**

1. Please indicate your background

*Options:*

Medical student, Resident, Fellow, Medical expert, Health care officer primary health care centre Resource Limited Country, Health care officer secondary health care centre Resource Limited Country, Health care officer tertiary health care centre Resource Limited Country, Nurse / Nurse practitioner, Other, please specify

**Country**

1. Which country are you currently living in?

**Format and Content***

1. The online format was appropriate for the subject matter and I was able to access all components of the activity without difficulty. Please note any concerns in the "Suggestions for improvement" field on the last page of this survey. *
2. The material was organized clearly for learning to occur. *
3. The content of this chapter/case is interesting to me. *
4. After studying this chapter/case I feel motivated to learn more on the subject. *

**Professional development***

This activity will assist in the improvement of my:

1. Competence
2. Performance
3. Patient Outcomes

**Level of difficulty***

1. The content and questions align with my knowledge level.

**Valuation****

1. The content and questions are:

**Miscellaneous***

1. I like self-assessment with multiple-choice questions.
2. The feedback that is given after answering the questions is appropriate.
3. I would like a few (more) open questions where answers are provided as a bullet list with relevant items. *
4. I would like a few (more) open questions for classroom/teacher feedback.

**Suggestions for improvement**

1. Please provide us with feedback regarding content, user interface, or other issues.

- Free Text

* Five-level Likert scale ranging from strongly agree (Likert scale =1), agree (Likert scale =2), neutral (Likert scale =3), disagree (Likert scale =4) and strongly disagree (Likert scale =5)

** Five-level Likert scale: too easy (Likert scale =1), easy (Likert scale =2), approtpriate (Likert scale =3), difficult (Likert scale =4) and too difficult (Likert scale =5)
